# William Twining

**Published:** 1836-04

**Authors:** 


### WILLIAM TWINING, ESQ.

*First Assistant Surgeon of the General Hospital, Calcutta.*

IN the premature death of this gentleman also, which took place on the 25th of August last, at Calcutta, we have to deplore an irreparable loss to our profession, more especially in the department of Oriental medicine. Fortunately for the science of which he was so zealous and successful a cultivator, he lived long enough to bring to comparative perfection the work in which he has embodied the results of his observation and experience in the pathology and treatment of the diseases of India. His "Clinical Illustrations of the more important Diseases of Bengal, with the result of an Inquiry into their Pathology and Treatment," published shortly before his death, in a second edition, in two volumes octavo, greatly enlarged and improved, will for ever remain a monument of his industry and sound pathological and practical knowledge; and will be long the faithful guide of the practitioner in India, until experience has unfolded to him the peculiarities of the diseases of that country. We hope to give a review of this valuable work in our next number, the first edition of which appeared in 1832.

Mr. William Twining appears to have been born in the British colony of Nova Scotia: it is at least certain that he passed his early years there, having served his apprenticeship to Dr. John Halliburton, a practitioner of Halifax, in that country. Having completed his education in London, and obtained the diploma of the College of Surgeons, he entered into the medical department of the army, on the 6th February, 1812. After being employed for some time at the military hospital at Hilsea, he proceeded to the Peninsula, where he served a short time, and again returned to Hilsea, where he remained until May, 1815, when he joined the British army in the Netherlands. He remained attached to the Army of Occupation until it returned from France, in December, 1818. Mr. Twining remained in England for three years, doing duty at Chatham, Maidstone, and the Isle of Wight, until September, 1821, when he embarked for Ceylon. He remained in Ceylon for a considerable time, and left the island to accompany His Excellency, Sir Edward Paget, to India. He was placed on the half-pay of the army at his own request, on the 25th Dec. 1823, and finally resigned his commission in the British service, by accepting a commutation allowance on the 7th Dec. 1830.

Mr. Twining, on retiring from the active service of the army, entered into private practice in Calcutta, where he was greatly esteemed. As one of the principal surgeons of the civil hospital, he had great opportunities of prosecuting his pathological and practical researches; and as secretary, and one of the most active members of the Medical Society of Calcutta, he had the means of extending his knowledge and the influence of his example throughout the profession in India. He was the author of many valuable papers in the Transactions of this excellent Association.

---

